# Function and Regulation Domains of a Newly Isolated Putative β-Actin Promoter from Pacific White Shrimp

**DOI:** 10.1371/journal.pone.0122262

**Published:** 2015-04-02

**Authors:** Yingli Shi, Marcus Soderlund, Jianhai Xiang, Yuanan Lu

**Affiliations:** 1 Department of Public Health Sciences, University of Hawaii at Manoa, Honolulu, Hawaii, United States of America; 2 Key Laboratory of Experimental Marine Biology, Institute of Oceanology, Chinese Academy of Sciences, Qingdao, Shandong, China; 3 University of Chinese Academy of Sciences, Beijing, China; International Centre for Genetic Engineering and Biotechnology, ITALY

## Abstract

Current development of transgenic shrimp research has been hampered due to the lack of the suitable promoters and efficient transfection methods for crustaceans. A 1642 bp sequence, containing 5’-upstream sequence, exon 1, intron 1 and partial exon 2, which is responsible for transcriptional initiation of the newly reported shrimp β-actin (*actinT1*), has been isolated from the Pacific white shrimp (*Litopenaeus vannamei*) and named as SbaP. To determine its function and potential application in marine biotechnology, the sequence and functional domains were examined by constitutive expression of the luciferase reporter gene. We have identified 5’ regions that play a central role in the expression of the β-actin gene. The proximal promoter (-1642/-1325) contains two highly conserved transcriptional sites, CCAAT box and CArG motif. Two negative (-1140/-924, -222/-21) and one positive (-810/-425) regulatory elements have been identified in intron1. Transient transfection assay with a construct containing proximal promoter and enhancer (SbaPΔ-222/+1Δ-1325/-924) regions of the shrimp β-actin coupled with luciferase and *EGFP* (enhanced green fluorescent protein) showed that the promoter was not only functional in sf21 cells, but promoter activity was more than 8-fold higher than a viral-origin promoter (ie1, white spot syndrome virus immediate early gene promoter). Furthermore, SbaPΔ-222/+1Δ-1325/-924 drove a successful expression of luciferase injection assay in vivo injection and also showed higher promoter activity than the ie1 promoter, suggesting that the expression vectors constructed with SbaPΔ-222/+1Δ-1325/-924 have important potential in gene transfer studies for shrimp and other crustacean species.

## Introduction

The Pacific white shrimp (*Litopenaeus vannamei*) is an aquaculture species found worldwide that accounted for 73% of total shrimp aquaculture production and generated the highest commercial value (USD 13.5 billion) in 2012 [1]. However, over the past 20 years, shrimp farming has suffered enormous losses with an estimated amount of 1 billion USD per year due to infectious diseases [2]. More understanding of transgenic techniques and their development are urgently needed to overcome shrimp diseases and reduce related economic loss. For example, studying gene regulation and its function in vivo and producing pathogen-resistant strains of shrimp is an essential step. Gene transfer techniques have been applied to several farmed shrimp species including Pacific white shrimp (*Litopenaeus vannamei*) [3], Chinese shrimp (*Penaeus chinensis*) [4], and black tiger shrimp (*Penaeus monodon*) [5]. Despite these successful studies, an essential step needed in the development of transgenic shrimp is to identify and use a suitable regulatory element adapted to the characteristics of shrimp to drive interesting gene expression. To date, most expression vectors are constructed using heterologous promoters in the transgenic shrimp research, such as the ie1 viral promoter [6], human cytomegalovirus promoter/enhancer [7], carp β-actin promoter [8], and EF-1α promoter [5]. However, there is an increasing demand for the development of the expression vectors constructed with homologous regulatory elements. Several studies have shown that although the heterologous gene constructs could be integrated into the host genome during expression in plant [9], mice [10], and fish [11], regulatory elements of fish origin were determined to be more efficient in transgenic fish than those from mammalian or viral origin [12, 13]. Present evidence from transgenic animal studies have indicated the importance and necessity for the development of transgenic shrimp using genetic materials originated from shrimp species. This is particularly important for promoting transgenic shrimp in commercial food markets since it may be easier for customers to accept.

β-actin promoters have been reported to be efficient ubiquitous regulatory elements and are widely used in transgenic mammalians [14] and fish [15]. However, the isolation and use of suitable promoters from crustaceans, including shrimp, has not yet been reported. In continuation of our previous description of the isolation and identification of two genomic sequence *actinT1* and *actinT2* from the Pacific white shrimp [16], we report here the molecular cloning of 5’-flanking sequence of *actinT1*, which includes 5’-upsteam sequence, 5’-untranslated exons, and 1^st^ intron. To establish the potential role of regulatory element in gene expression, functional analysis of the newly obtained shrimp promoter sequences were conducted. The results show that there are three regulatory elements in the 1^st^ intron. Furthermore, to our knowledge, this is the first report where the sequence in the 3’-distal of 1^st^ intron could also significantly suppress the promoter activity.

## Materials and Methods

### Experimental Animals

Specific pathogen-free (SPF) Pacific white shrimp (*Litopenaeus vannamei*), were obtained from Oceanic Institute, Oahu, Hawaii, and reared in aerated fresh seawater at an ambient temperature of 20–22°C at 20 ppt salinity. Natural seawater was obtained from Waikiki aquarium, Oahu, Hawaii and used in all experiments. The animals were kept in 20-gallon tanks for 5 days to acclimatize, then were used for experimental tests.

### DNA extraction

250 mg of tail-muscle tissue were collected from juvenile Pacific white shrimp, *L*. *vannamei*, ground with a pre-cooled mortar and pestle under liquid nitrogen and digested in 3.0 mL of proteinase K solution (150 mM NaCl; 10 mM Tris-HCl, pH 7.4; 0.1 mM EDTA, pH 8.0; 0.08% SDS; 500 μg/mL proteinase K; 50 μg/mL RNase A) at 56°C for 3 hours. Genomic DNA was extracted by the conventional phenol/chloroform method [17].

### Inverse PCR (iPCR) and DNA sequencing

1 mg of shrimp DNA was digested with Nla III and then self-ligated with T4 DNA Ligase (New England Biolabs, NEB, MA, USA). Forty nanograms of the DNA were used as a template in a 25.0 μL iPCR reaction using primers indicated in Table 1. Nested iPCR was performed with the use of 5.0 μL amplicon of first-round PCR amplification as a template for the second round. The resulting iPCR product was gel purified using the QiaQuick Gel-Extraction Kit (Qiagen, CA, USA) and sequenced using a capillary-electrophoresis based automatic DNA sequencer with fluorescent BigDye terminator chemistry (Applied Biosystems).

**Table 1:** List of primer pairs used in iPCR.

Primer name	Primer Sequence
iPCR inr Nil Se	CGCCCTCACGAACCTACCG
iPCR inr N i1 As	AGAACTTGGACGAATGGGAGGCTA
iPCR out i1 Se	ATTCGCCTAAACTCCGCCCTCACG
iPCR out i1 As	TATTTTGGGTCACGATTGGGGTCTCAC

PCR conditions: initial 3 minute 95°C denaturation, followed by 37°C of 1 min at 95°C, 30 seconds at 54°C, 4 minutes at 72°C, with a 10 minute final extension at 72°C.

### Bio-informatics analysis

Inverted PCR-generated new sequences, named as SbaP, were aligned with previously reported 5’ UTR and coding sequences of the β-actin gene (*actinT1*) to determine the orientation of identified shrimp sequences. Both BLASTn (Altschul) search and manual sequence analysis of the putative promoter region and 1^st^ intron sequences were performed to identify significant matches and conserved motifs of GenBank sequences. Chromas software (Technelysium Pty Ltd), ClustalW and BLAST were used to analyze and align the electropherograms and sequence generated by the ABI basecaller software. The transcription elements (TEs) in the 5’-flanking sequences were investigated through the TFSEARCH system (http://www.cbrc.jp/research/db/TFSEARCH.html, Accessed March, 2014) and the core promoter sequences were predicted by the Neural Network Promoter Prediction method using default parameters (http://www.fruitfly.org/seq_tools/promoter.html, Accessed March, 2014).

### Cell culture

Insect *Spodoptera frugiperda* Sf21 cells (manufactured by Invitrogen) were routinely cultured in TNM-FH medium (Invitrogen, NY, USA) supplemented with 10% fetal bovine serum (FBS, GE Healthcare, WI, USA) and grown at 27°C.

### Construction plasmid for luciferase assay

To evaluate the promoting activity and regulatory element of SbaP, a promoter-luciferase cassette was constructed. All primers used to amplify specific fragments by PCR method are listed in Table 2. PCR products were ligated into pCR2.1-TOPO vectors (Invitrogen, NY, USA) and then subcloned into the Sac I/Bgl II site of pGL4.10 [*Luc2*] vector (Promega, CA, USA). The serial-deleted fragments of SbaP were constructed using the restriction enzymes listed in Table 3. The plasmid pGL-SbaP was digested by specific enzymes, blunted end by DNA polymerase I, Large (Klenow) Fragment, self-ligated using T4 ligase and then transformed into DH5-alpha competent cells (NEB, MA, USA).

**Table 2:** List of primer pairs used in construction, the restriction enzyme sites were underlined.

Primer name	Primer Sequence	Length (bp)	Tm (°C)
5’-upstream (SacI) F	CTCGAGCTCACTAGTAACGGCCGCCAGTGTG	585	62
5’-upstream (BglII) R	TTAAGATCTACTTGGACGAATGGGAGGCT		
5’-UTR and intron (SacI) F	TTTGAGCTCCGTCCGCCCTTTGTAAGTAT	1175	58
5’-UTR and intron (BglII) R	TGCAGATCTCGGCTTCTTGTTGTTGTTGT		
5’-full sequence (SacI) F	TTTGAGCTCAAAATGAGGCGGCGGCAATG	1642	58
5’-full sequence (BglII) R	TGCAGATCTCTTGTTGTTGTTGTTTTAC		
Ie1 (EcoRV) F	TTGTTCGATATCATGTGGCTAATGGAGAATTGT	504	57
Ie1 (BamHI) R	TTTTGGATCCCTTGAGTGGAGAGAGAGAGCT		
Polh F	AGACGCACAAACTAATATCACAAACTGGA	305	53
Polh R	CGTGTCGGGTTTAACATTACGGATT		

**Table 3:** List of restriction enzymes used in the serial-deletion.

Constructs	Enzyme1	Enzyme 2
pGL-SbaPΔ-1642/-1325	KpnI	NsiI
pGL-SbaPΔ-1325/-924	NsiI	XmnI
pGL-SbaPΔ-222/+1	EcoRV	EcoRV
pGL-SbaPΔ-425/+1	AclI	EcoRV
pGL-SbaPΔ-810/+1	PpuMI	EcoRV
pGL-SbaPΔ-924/+1	XmnI	EcoRV
pGL-SbaPΔ-222/+1Δ-1325/-924	EcoRV	NsiI/XmnI
pGL-SbaPΔ-222/+1Δ-924/-810	EcoRV	XmnI/PpuMI
pGL-SbaPΔ-222/+1Δ-810/-425	EcoRV	PpuMI/AatII
pGL-SbaPΔ-222/+1Δ-1642/-425	EcoRV	KpnI/AatII
pGL-SbaPΔ-222/+1Δ-1325/-425	EcoRV	NsiI/AatII
pGL-SbaPΔ-1325/+1	NsiI	EcoRV
pGL-SbaPΔ-222/+1Δ-1325/-810	EcoRV	NsiI/PpuMI

Note: the position of translation start site ATG is defined as +1.

### Construction of EGFP plasmid

To explore the relative strength of promoter activity, the sequences of SbaP, SbaPΔ-222/+1Δ-1325/-924, *ie1* (WSSV immediate early gene) promoter, and *Polh* (Polyhedrin) promoter were cloned into the EGFP (enhanced green fluorescent protein) reporter vector pGL-EGFP, respectively, in which the luciferase gene was replaced by *EGFP*. The *ie1* promoter of the white spot syndrome virus (WSSV) was cloned from WSSV genomic DNA and used as a positive control. The *Polh* promoter was also cloned from baculovirus genomic DNA (Table 2). Plasmids for transfection were prepared using a plasmid mini kit (Omega Bio-Tek, GA, USA).

### Transfection and luciferase assay

Transfection experiments were performed in 96-well culture plates. Briefly, one hour before transfection, recipient cells were seeded into wells at a density of 3.8×10^4^ cells/ well. For dual-luciferase assay, the cells in 96-well plates were co-transfected with 500ng of promoter-luciferase constructs and 25ng pRL-tk vector (Renilla luciferase gene driven by HSV-thymidine kinase promoter) using Profectin transfection reagent (AB vector, CA, USA), according to the manufacturer’s protocol. At 12 hours post transfection, the transfection mixture was replaced with the medium containing 10% FBS. Two days after transfection, cells were lysed and the activities of firefly and Renilla luciferase were measured using dual-luciferase reporter assay (Promega, CA, USA) according to the manufacturer’s instruction and chemiluminescence was read by a 1420 multilabel counter (PerkinElmer). Each experimental test was carried out in triplicate.

For expression of EGFP, sf21 cells were transfected with 200 ng of reporter construct DNA in 100 μL medium per well using 1 μL FuGENE transfection reagent (Promega, CA, USA) according to the manufacturer's recommendations. At 48 h post transfection, EGFP expression was evaluated using an inverted fluorescence microscope (Nikon).

### In vivo expression of luciferase

To test whether SbaPΔ-222/+1Δ-1325/-924 could drive the expression of reporter gene (*Luc*) in *L*. *vannamei*, plasmid pGL-SbaPΔ-222/+1Δ-1325/-924, pGL-ie1, pGL-SbaP and pGL4.10 [*Luc2*] were injected into shrimp separately. Test shrimp were divided into 4 groups according to plasmids injected (6 individuals in each group): GL; SbaP; SbaPΔ-222/+1Δ-1325/-924 and ie1, and kept in a separate tank. The injection was performed intramuscularly at the third abdominal segment. Each shrimp was injected with a dose of 20 μg plasmid (based on the result of preliminary experiments) in a total volume of 50 μL with PBS.

To access in vivo expression of luciferase at the transcriptional level, real-time RT-PCR was performed. At 48 h post injection, muscles from each shrimp were collected separately, and pulverized in liquid nitrogen. 0.1 g grinded tissue of each individual was used for RNA extraction by using TRIzol (Invitrogen, NY, USA) by following the manufacturer’s instructions. DNA contamination was removed by using RNase free DNase I (Sigma-Aldrich, MO, USA) and synthesis of cDNA was performed with iScript reverse transcription supermix (Bio-Rad, CA, USA) according to manufacturer’s protocol. The transcription level of luciferase was detected using the forward primer RT-LucF (5’TGCAGTTCTTCATGCCCGTGTTG3’) and reverse primer RT-LucR (5’ TTTGCAGCCCTTTCTTGCTCACG3’) (147 bp product). As a stably expressed reference gene, 18s rRNA was also detected using forward primer RT-18SF (5’ TATACGCTAGTGGAGCTGGAA3’) and reverse primer RT-18SR (5’ GGGGAGGTAGTGACGAAAAAT3’), which resulted in an expected PCR fragment of 136 bp in length. Each cDNA sample was amplified in triplicate along with the internal control gene when analysed by real-time PCR and all reactions were run in an iQ5 Multicolor Real-Time PCR Detection System (Bio-Rad, CA, USA). The real-time RT-PCR reaction was carried out with iQ SYBR Green supermix (Bio-Rad, CA, USA), 0.3 μM each forward and reverse primer and 1 μL cDNA template. The PCR parameters consisted of 1 cycle of 95°C 2 min; 40 cycles of denaturation at 95°C for 15 s, annealing and extension at 60°C for 1 min, respectively. A melt cycle, in which the PCR product was denatured from 65°C to 95°C, was added to the thermal profile to produce dissociation curve.

The expression of luciferase was normalized to 18s rRNA, Ct values of amplified target gene (Ct_Luc_) and internal control gene (Ct_18s_) of each sample was calculated by iQ5 software (Bio-Rad), ΔCt value was calculated using formula ΔCt = Ct_Luc_- Ct_18s_, to normalize the data, ΔΔCt was produced by subtracting ΔCt of each test sample from the average ΔCt of the calibrator (pGL-SbaP injected to shrimp). Then the relative expression of luciferase at transcription level of group ie1 and SbaPΔ-222/+1Δ-1325/-924 relative to group SbaP was calculated with formula 2^-ΔΔCt^.

### Statistical Analysis

The luciferase assay results are presented as mean ± S.D. and were compared between groups by one-way analysis of variance (ANOVA) followed by Bonferroni’s multiple comparison tests. The value was considered to be statistically significant at P<0.05.

## Results

### Isolation and sequence analysis of 5’-flanking sequences of shrimp β-actin

**Fig 1:**
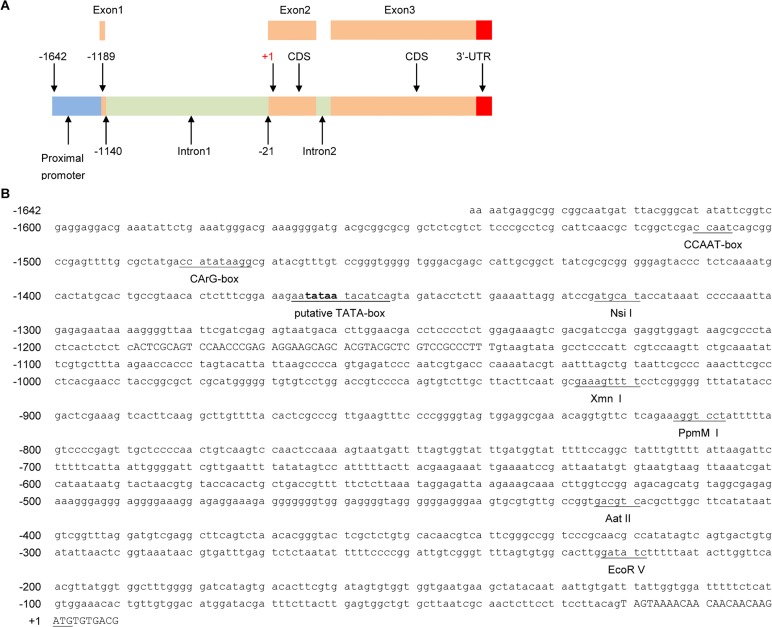
Nucleotide sequences of the fragment of a beta-actin gene isolated from the shrimp, *Litopenaeus vannamei*. A: A schematic map of the genomic sequence of L.v. beta-actin and the 5’-flanking sequences. The numbers indicate the positions (the first base of starting codon ATG was set as position+1). B: sequence of β-actin gene upstream region, untranslated exon1, intron1 and partial exon2. Sequences in exons are shown in uppercase letters, whereas those in introns and flanking regions are shown in lowercase letters. Numbering of the nucleotide sequences is given at the left. Underlined boldface letters shows CAAT, CArG and TATA boxes in the 5’-flanking region. The useful restriction enzyme sites are underlined with their name.

We have previously reported the isolation of a 1,128-bp *actinT1* gene from the Pacific white shrimp, *L*. *vannamei*, by screening a cDNA library prepared from shrimp eyestalk [16]. To further study the shrimp actin initiation of transcriptional promoting sequence and regulatory region, a 1, 642-bp of 5’-flanking sequence was isolated and sequenced from the shrimp genomic DNA by inverse PCR (Fig 1B).

Alignment of 5’-flanking sequence was carried out using the sequences previously obtained from the cloning of actinT1. The result indicated that this newly isolated 1.6 kb of sequences containing a proximal promoter, the untranslated exon1 and partial exon2, intron1 (Fig 1A), and sequences at exon-intron boundary regions were well consistent with the GT-AG splicing rule [18]. Although the coding region of actinT1 shows high identities with other species, the sequence of the promoter region and 1^st^ intron show no close homologies to actin from other species using a BLASTn search. The transcription elements (TEs), a CCAAT and a TATA box were predicted using the TFSEARCH system. The GenBank accession number of the 5’-flanking sequence is KJ850951.

### Analysis of the proximal promoter regions

**Fig 2:**
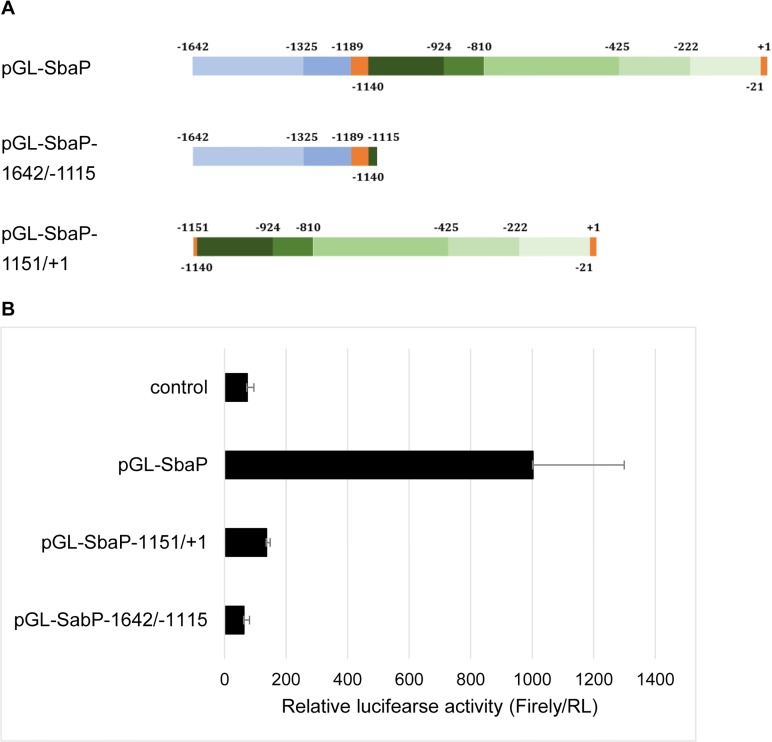
Examination of activity of 5’-upsream sequences and 1^st^ intron of the shrimp β-actin gene based on a reporter assay. A: schematic diagram of promoter region of luciferase reporter gene constructs. Showing various 5’-flanking sequence sequences of shrimp β-actin gene fused with luciferase gene. B: the relative levels of reporter gene expression in sf21 cells are shown. The constructs were transiently co-transfected into cells along with pRL-tk control vector. The activity of firely and Renilla luciferase in the cell lysate were measured using Dual-luciferase reporter assay (Promega) at 48h post transfection. Firefly luciferase activity was normalized to Renilla luciferase activity. The Bars indicated mean ±S.D. of luciferase activity (n = 3).

To explore the promoter activity of newly isolated shrimp β-actin sequence, several constructs containing various regions of the 5’-flanking sequence were generated and fused to a luciferase gene. An initial simple construct contained all 1, 642 bp of 5’-flanking sequences (construct named pGL-SbaP, Fig 2A), which was compared with the luciferase reporter constructs composed of proximal promoter (pGL-SbaP-1642/-1115) and 1^st^ intron (pGL-SbaP-1151/+1), respectively. Since cell lines derived from shrimp were not available, most of the current promoter studies were carried out under xenogenic conditions [19, 20]. The result indicated that the proximal promoter was able to drive luciferase gene expression successfully (Fig 2B). However, it showed a severe reduction compared to that of 5’-flanking sequence (pGL-SbaP), indicating there are regulatory elements within the 1^st^ intron.

### Testing of shrimp beta-actin gene regulatory regions

**Fig 3:**
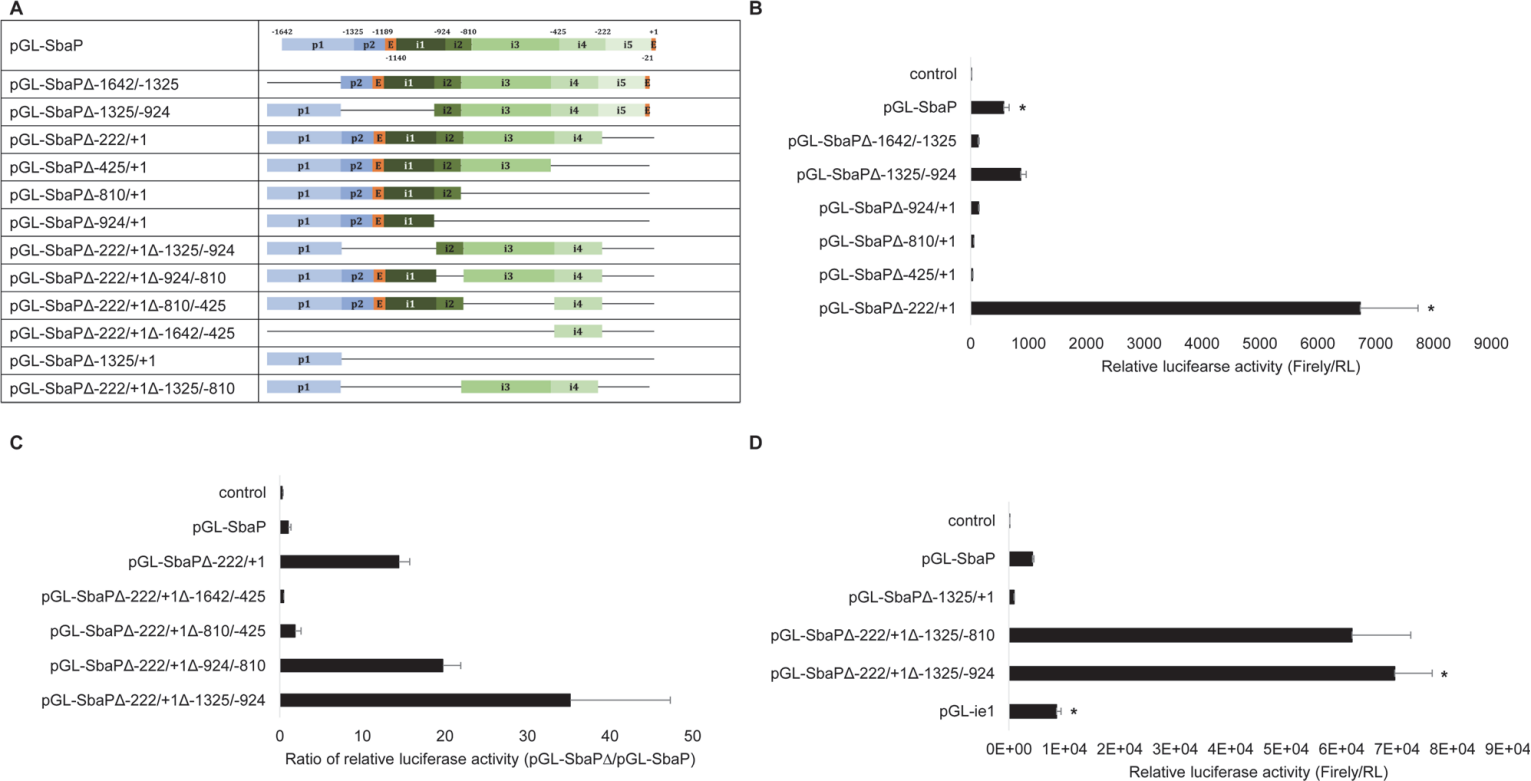
Characterization of regulatory region of 5’-flanking sequences of the shrimp β-actin gene based on the reporter assay. A: schematic diagram of series deletion constructs with luciferase reporter gene, which were made as described in Materials and Methods. Δ indicates a deletion; B-D: the relative levels of reporter gene expression in sf21 cells are shown. The constructs were transiently co-transfected into cells along with pRL-tk control vector. The activity of firely and Renilla luciferase in the cell lysate were measured using Dual-luciferase reporter assay (Promega) at 48h post transfection. Firefly luciferase activity was normalized to Renilla luciferase activity. The Bars indicated mean ±S.D. of luciferase activity (n = 3). *indicates statistical significance (P<0.01).

To explore the regulatory element of the shrimp β-actin gene in the 1^st^ intron region, a series of deletion mutants within the 1.6-kb upstream sequence coupled to luciferase were constructed. The deletion mutants were based on pGL-SbaP, and the deletion was performed according to the restriction endonuclease sites in the SbaP (Fig 3A). By transfecting these constructs transiently into insect sf21 cells, the results indicated that with the deletion of either proximal promoter (pGL-SbaPΔ-1642/-1325) or sequence located at -425/+1 (pGL-SbaPΔ-425/+1, pGL-SbaPΔ-810/+1 and pGL-SbaPΔ-924/+1), the luciferase activity dropped to less than 20% of that obtained with SbaP. However, constructs with deletion of the sequence located between -1325/-924 (pGL-SbaPΔ-1325/-924) and -222/+1 (pGL-SbaPΔ-222/+1) revealed an increased luciferase activity compared to SbaP, especially the 3’-distal region of the 1^st^ intron (pGL-SbaPΔ-222/+1), showing 10-fold up-regulation. Altogether, our results suggest that there is more than one domain affecting promoter activity of the putative shrimp β-actin promoter (Fig 3B).

These findings led to more extensive deletion studies to the pGL-SbaPΔ-222/+1 and the -425/-222 sequences. The results indicated that the luciferase activity of pGL-SbaPΔ-222/+1Δ-810/-425 dropped to 15% than that of pGL-SbaPΔ-222/+1, while the constructs with deletion of the sequences located on -1325/-810 had a fairly high luciferase activity (Fig 3C). The construct pGL-SbaPΔ-222/+1Δ-1642/-425 only exhibited low luciferase activity, which indicated that there is no any stronger alternative promoter in the intron and the high activity originated from the proximal promoter.

To verify the function of the sequence located at -924/-810 and evaluate the strength of promoter activity, the expression vector pGL-SbaPΔ-222/+1Δ-1325/-810 and pGL-ie1 were constructed. The promoter activity of SbaPΔ-222/+1Δ-1325/-810 was compared with that of SbaPΔ-222/+1Δ-1325/-924, SbaPΔ-1325/+1, SbaP and ie1. The results showed that there was no statistically significant difference in promoter activity between pGL-SbaPΔ-222/+1Δ-1325/-810 and pGL-SbaPΔ-222/+1Δ-1325/-924 (p>0.05), which indicates that the sequence located at -924/-810 is a non-essential part for the promoter activity (Fig 3D). Among these constructs, SbaPΔ-222/+1Δ-1325/-924 expressed the strongest promoter activity, which is 75-fold higher than that of the proximal promoter containing no enhancer (SbaPΔ-1325/+1), 16-fold higher than that of original SbaP, and even 8-fold higher than that of the viral promoter ie1. The detailed data of luciferase assay results were provided in S1 Dataset.

### Expression of EGFP using the shrimp β-actin gene promoter/enhancer in sf21 cells

**Fig 4:**
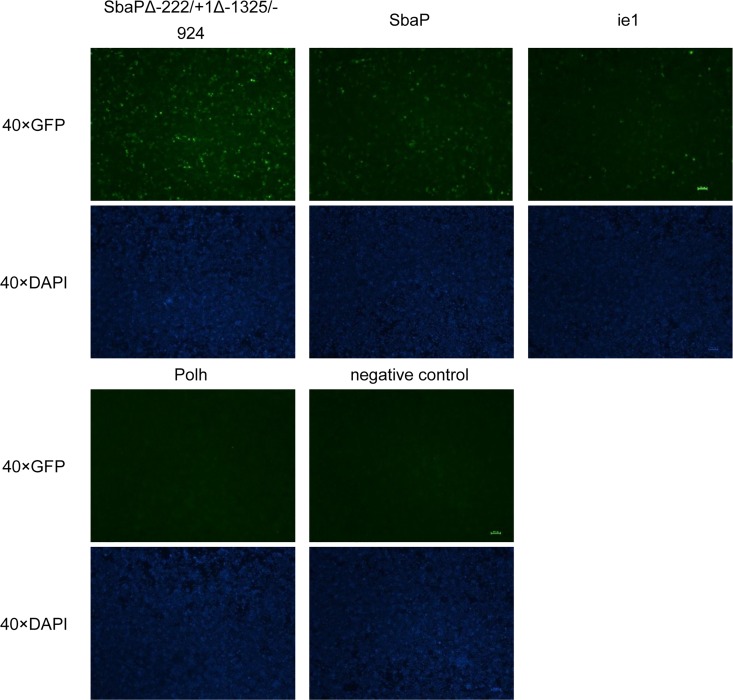
Expression of EGFP driven by SbaPΔ-222/+1Δ-1325/-924 in sf21 cells. Sf21 cells were transfected with pGL-SbaPΔ-222/+1Δ-1325/-924-EGFP (a, f), pGL-SbaP-EGFP (b, g) and pGL-ie1-EGFP (c, h), pGL-Polh-EGFP (d, i) and pGL (e, j) were observed under a fluorescence microscope at day 2 post transfection. The green fluorescence protein gene (EGFP) can be detected in a, b, c (positive control), but not in d and e (negative control). The nuclei were stained with DAPI dye. Bar = 100 μm.

Three regulatory elements in the 1^st^ intron region of shrimp β-actin were identified according to serial deletion studies (Fig 3). For further understanding the shrimp β-actin promoter on a transcriptional level, another reporter gene *EGFP* was employed to examine the newly isolated β-actin promoters and its derived constructs with varied sequence deletion, together with ie1 and Polh to be subcloned into a promoter-EGFP cassette. Among all these constructs, promoter/enhancer (SbaPΔ-222/+1Δ-1325/-924) exhibited the highest expression of EGFP (Fig 4), and the robust expression of the EGFP reporter gene was observed at day 2 post transfection.

### In vivo verifying the promoter activity of shrimp β-actin gene promoter/enhancer

**Fig 5:**
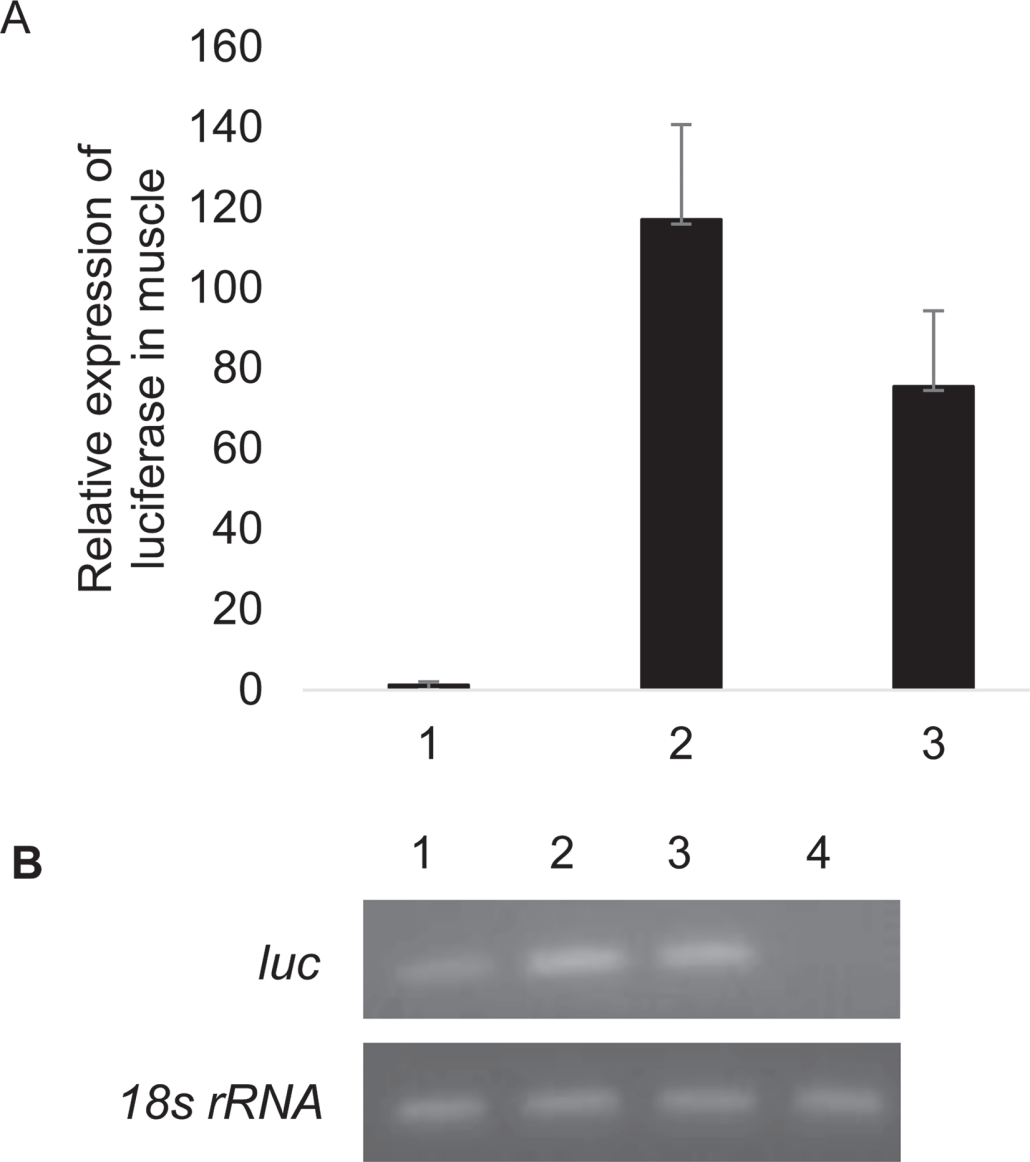
Expression of Luciferase in vivo at day 2 post injection. (A) Relative transcription of luciferase in muscle after different group injection. 1: SbaP group; 2: SbaP (Δ-222/+1Δ-1325/-924) group; 3: ie1 group. (B) Real time RT-PCR products. The products were run in 2% agarose, products in upper and lower line were amplified using detection primer pairs (RT-LucF/R) and internal control primer pairs (RT-18SF/R) respectively. 1: SbaP group; 2: SbaP (Δ-222/+1Δ-1325/-924) group; 3: ie1 group; 4: negative control, pGL group.

After functional analysis of proximal promoter and regulatory elements of SbaP in sf21 cells, the plasmid constructs (pGL-SbaP, pGL-SbaPΔ-222/+1Δ-1325/-924, pGL-ie1 and pGL) were injected into shrimp to verify and evaluate promoter activity in vivo. Shrimp muscle tissue was collected at day 2 post injection and real time RT-PCR was performed to detect the expression of luciferase gene at the transcriptional level. The result confirmed that SbaPΔ-222/+1Δ-1325/-924 could successfully drive reporter gene (*luc*) expression in vivo, and consistently expressed higher promoter activity than that of ie1 promoter (Fig 5).

## Discussion

Although several shrimp β-actin have been reported in literature [21–23], little is known about the functional characterization of their promoters. Since the study on homologous promoters will not only facilitate the transgenic research and analysis of functional genes, but also be beneficial for the generation of transgenic shrimp which can be readily available for FDA approval, the 5’-flanking sequence of shrimp β-actin (*actinT1*) was cloned, and its functional domains were analyzed and determined in this study.

**Fig 6:**
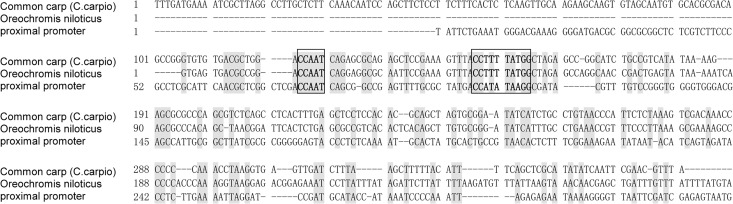
Putative-regulatory sequence of the β-actin gene homologues. Sequence alignment of the proximal promoter region is shown. Black and open boxes mark conserved sequences and known factor binding sites, respectively; CCAAT-box and CArG motif. GenBank accession number of compared fish β-actin genes are as follows: *Oreochromis niloticus* (AY116536.1); Common carp (*C*. *carpio*) (M24113.1).

Our study has determined that the sequence located at -1642/-1325 of this putative shrimp promoter is an essential domain for the promoter activity. Despite the sequence of 5’-flanking region showing no close homologies to actins from other species, a stretch of 327 bp of proximal promoter was compared with that of *Oreochromis niloticus* and Common carp (*C*.*carpio*), and a putative transcription factor binding sites, CCAAT was identified at the location of -322 bp from transcriptional start site (TSS). In addition, a highly conserved CArG (CC (A/T) _6_GG) motif was also identified at the location of -293 (Fig 6). The distances between the CCAAT and CArG boxes and the TSS are known to be variable, but they are usually within the first 220 bp from the Cap signal, upstream of the TATA box [24, 25]. In this study, a TATA box was predicted using the TFSEARCH system and was within the location of 140 bp. It has been proven that the CCAAT-box is an essential element for the promoter activity when joined to a heterologous gene [26] and CArG is an essential serum-response element positioned in the actin promoter region between the CCAAT and TATA box [27, 28].

The deletion of the partial proximal promoter and transcription start site (pGL-SbaPΔ-1325/-924) did not abolish the reporter gene expression (Fig 3B). This suggests that there is a cryptic transcription start site (TSS) when part of the proximal promoter is deleted. This phenomenon was in agreement with the study in the Mud loach and carp β-actin expression [29].

Previous studies indicated that the proximal promoter and the 1^st^ intron enhancer elements were required for the strong activity of the β-actin promoter in other species [30, 31]. A short sequence including a CArG motif in the distal region of the β-actin 1^st^ intron in humans and fish had positive transcriptional activity [15, 30, 31]. However, there are no evolutionarily conserved sequences [32] that exist in the 1^st^ intron of shrimp β-actin, and *Schistosoma mansoni* β-actin do not have the CArG box [33] in the 1^st^ intron as well, which is an obvious difference to that found in vertebrates.

**Fig 7:**
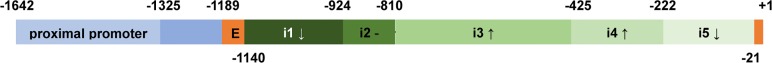
Summary of the shrimp β-actin gene regulatory regions. The regulatory loci identified in this report are designated by ↑ when positive, and ↓ when negative. The numbers indicate the positions (the first base of starting codon ATG was set as position+1).

To further characterize the regulatory element of the shrimp β-actin gene, we explored the contributions of five regions, i1 to i5, of the first intron with respect to their abilities to modulate gene expression (Fig 7). Firstly, i3 and i4 constitute a positive regulatory element located at -810/-222 of the 1^st^ intron. In particular, i4 (-425/-222) is an essential part for expression of the β-actin gene and the promoter (pGL-SbaPΔ-425/+1) activity was almost undetectable when this portion of the sequence was deleted. Similar results were observed in previous studies in fish [29], chicken [34], and humans [35]. Secondly, i2 (-924/-810) was a non-essential part for the promoter activity, as the deletion of i2 did not affect the promoter activity significantly (p>0.05). Thirdly, the i1 (-1140/-924), located in the 5’-region of the 1^st^ intron, was a negative regulatory element of 216 bp, which is similar to the previously reported carp β-actin promoter [30]. Lastly, i5 (-222/-21), a distal region of the 1^st^ intron, has shown a strong inhibitory effect of the promoter activity. This observation, to the best of our knowledge, has not been reported previously.

In summary, the sequences located at -1642 to -1325, including CCAAT and CArG motif, were identified as essential domains for promoter activity. Three regulatory elements including one enhancer (-810/-222) and two silencers (-1140/-924, -222/-21) were also detected in the 1^st^ intron. The sequence located at -425/-222 and -222/-21 was determined to affect promoter activity significantly. In the following transduction application, we removed two silencers and some non-essential sequences (-1325/-1141), then combined the rest of sequences together to generate a compact construct promoter/enhancer (pGL-SbaPΔ-222/+1Δ-1325/-924), which expressed relative strong promoter activity compared with SbaP. This newly constructed promoter/enhancer exhibited an enhanced promoter activity, which is much higher than the Polh and ie1 promoter reported previously as so-called strong promoter with high transcriptional activity in sf21 cells [36], and this finding also verified in vivo. Moreover, the shrimp β-actin gene is constitutively expressed in all shrimp tissues at all developmental stages, and thus, this shrimp β-actin promoter/enhancer (SbaPΔ-222/+1Δ-1325/-924) represents an ideal promoter for the construction of an expression vector in the application of gene transfer in shrimp and other crustacean species. The use of a β-actin promoter base-expression vector could be beneficial for establishing transgenic shrimp for specific expression of interested genes. This newly isolated SbaP is currently being tested and evaluated for its function in driving transgene expression in primary cultures of shrimp cells *in vitro* and also in its application for driving transgene expression and function in affected shrimp *in vivo*.

## Supporting Information

S1 Dataset The detailed data of luciferase assay results. Transfected cells in each well were lysed and the activities of firefly and Renilla luciferase were measured using dual-luciferase reporter assay, chemiluminescence was read three times. Each experimental test was carried out in triplicate.
(XLSX)
